# Association of periodontitis and tooth loss with extent of coronary atherosclerosis in patients with type 2 diabetes mellitus

**DOI:** 10.3389/fendo.2023.1243992

**Published:** 2023-11-23

**Authors:** Minhua Shen, Zhen Li, Huizhi Li, Xinfeng Yan, Bo Feng, Lei Xu

**Affiliations:** ^1^ Department of Stomatology, East Hospital, Tongji University School of Medicine, Shanghai, China; ^2^ Department of Endocrinology, East Hospital, Tongji University School of Medicine, Shanghai, China

**Keywords:** periodontitis, tooth loss, diabetes, coronary atherosclerosis, CAC scores

## Abstract

**Aim:**

The objective was to investigate the association of periodontitis and tooth loss with extent of diabetic coronary atherosclerosis.

**Materials and methods:**

272 patients who were hospitalized at Shanghai East hospital and underwent a coronary artery calcium (CAC) CT scan were enrolled in this study. Individuals were grouped based on their CAC scores into a normal-to-mild coronary atherosclerosis (AS) group (0 ≤ score ≤ 100, n=184) and a moderate-to-severe group (score≥101, n=88). Periodontitis parameters and number of missing teeth were evaluated for every patient. The severity of periodontitis was categorized as mild, moderate, or severe. The taxonomic composition of the microbiota was determined using full-length 16S ribosomal RNA gene sequencing. Salivary inflammatory factors were tested by ELISA.

**Results:**

Clinical attachment loss (CAL) (P =0.05) and the number of teeth lost (P = 0.016) were significantly higher in the moderate-to-severe coronary AS group, with these differences being more obvious in younger patients and patients with short-duration diabetes. Multivariate logistic regression analysis revealed that CAL (OR = 1.231, 95% CI = 1.066–1.214, P = 0.047) and having 10–19 missing teeth (OR = 1.604, 95% CI = 1.393–6.555, P = 0.05) were strongly associated with the presence of moderate-to-severe coronary AS. Salivary IL-6 and TNF-α levels, as well as levels of Porphyromonas gingivalis and Neisseria mucosa, were significantly elevated in the moderate-to-severe coronary AS group.

**Conclusion:**

It was found that both tooth loss and CAL were related to the extent of diabetic coronary AS. Saliva inflammatory factors and oral bacteremia may be new biomarkers for moderate-to-severe coronary AS.

## 1 Introduction

Diabetes mellitus is a risk factor for both atherosclerosis and periodontal disease (1). Cardiovascular diseases (CVDs) are the primary cause of mortality and morbidity in diabetes. Diabetes is unquestionably the most important risk factor for CVDs (2). Numerous epidemiological studies have identified a high degree of association between diabetes and periodontal disease, and periodontal diseases have even been proposed as a sixth complication of diabetes (3). The past decade has produced a growing number of investigations surrounding the idea that infection and inflammation occurring as a result of oral pathogenesis can lead to the progression of systemic disease (4–6). It is believed that infection-mediated upregulation of cytokines and other inflammatory mediators plays a central role in this pathologic process (7–9). The high prevalence of CVDs and periodontitis in individuals with diabetes may be attributed to an increased inflammatory response leading to atherosclerosis; this response is usually more extensive and develops at an earlier age in individuals with diabetes compared to those without diabetes (10).

Periodontal diseases are a group of common chronic infections that produce an exaggerated inflammatory response to the oral pathogenic microflora. Periodontitis is the main category of periodontal diseases. It affects the attachment of connective tissue and the supporting bone around the teeth, leading to tooth mobility and subsequent tooth loss (11, 12). Since an initial report in 1989, several further case–control studies, cross-sectional studies, and cohort studies have demonstrated a positive and independent correlation between periodontitis and CVDs (13–16). It should be noted that the specific criteria for periodontitis and CVDs used in these studies have varied greatly. This diversity may account for some of the variability seen among studies in terms of the strength of association between these two conditions. Although no clear explanation for the association between periodontitis and CVDs has been presented so far, there are several proposed mechanisms, both direct and indirect, accounting for this relationship (17–19). Periodontal bacteria and their products may enter systemic circulation (20, 21), inducing platelet aggregation, foam-cell formation (22), and the development of atheroma (a direct mechanism) (23, 24). As a result of the host-mediated immune response to this microbial challenge, serum concentrations of inflammatory mediators, including interleukin-1β (IL-1β), tumor necrosis factor-α (TNF-α), and prostaglandin E2, which are elevated in periodontal disease (25). These mediators trigger atherosclerosis through recruitment of inflammatory cells to blood vessels, proliferation of vascular smooth muscle cells, and induction of vascular fat degeneration and intravascular coagulation (an indirect mechanism) (26).

There is strong evidence for a link between oral health and cardiometabolic health. Recent evidence suggests that exposure to the adverse oral microbial communities that underlie periodontitis might also contribute to the etiology of cardiometabolic diseases, including diabetes (27–29). However, few studies have investigated the relationship between oral microbial communities and the extent of diabetic coronary atherosclerosis. The identification of microbial signatures that emerge early in disease could help to elucidate the potential shared microbial etiology of periodontitis and cardiometabolic disease and provide biomarkers that can aid in recognition of severe coronary atherosclerosis (AS) in patients with type 2 diabetes mellitus (T2DM).

Although studies have reported separately on associations between periodontitis and diabetes (30, 31) and between periodontitis and coronary heart disease (32, 33), the impact of periodontitis on coronary AS in the presence of diabetes is not well delineated. It is remains unclear whether the relationship between periodontitis and coronary AS in patients with T2DM has its own characteristics, and how the duration of diabetes or the level of blood glucose control can affect this relationship. Previous studies have mainly examined the association between periodontitis and myocardial infarction risk, CVD mortality, fatal coronary events, or incidence of CVDs. It remains unclear whether a clinically significant association exists between the periodontitis and the extent of coronary atherosclerosis. Measurement of coronary artery calcium (CAC) score using non-contrast cardiac gated computed tomography provides a successful and simple scoring algorithm for quantification of CAC and estimation of the extent of coronary artery disease per atherosclerotic lesion (34). In the present study, we have chosen CAC score as an indicator of the extent of coronary AS. Moreover, few studies have focused on the relationship between changes in salivary inflammatory factors or oral microflora and coronary AS in patients with T2DM. However, it should be noted that epidemiological research cannot identify the cause of such a relationship. Therefore, there is a need for studies evaluating the relationship between periodontitis and extent of coronary AS in patients with T2DM and providing evidence to support the identification of moderate and severe coronary artery disease in dental clinics.

In this article, we present cross-sectional data on (1) the relationship of clinical measures of periodontitis and tooth loss with the extent of diabetic coronary AS, and (2) the changes in salivary inflammatory factors and oral microbial communities that occur in patients with varying degrees of diabetic coronary AS.

## 2 Materials and methods

### 2.1 Study participants

A total of 272 patients with T2DM, all of Chinese Han ethnicity, who were hospitalized at the Department of Endocrinology, Shanghai East Hospital Affiliated to Tongji University, from November 2020 to December 2021 were included in our study. Patients were diagnosed with T2DM according to the 1999 World Health Organization (WHO) criteria. We excluded patients with type 1 diabetes mellitus or specific types of diabetes mellitus, acute complications of diabetes, liver or renal dysfunction, serious cardiac arrhythmias or acute cardiac insufficiency, diabetic foot ulcers, or history of acute myocardium infarction, percutaneous coronary intervention, cerebral infarction, tumor, or psychosis. We obtained written informed consent from all of the participants. The study was approved by the Human Research and Ethics Committee of Shanghai East Hospital and adhered to the tenets of the Declaration of Helsinki.

### 2.2 Data collection and physical assessment

Participants’ demographic details, frequency of dental visits, education level, and medical history were carefully recorded. Medical histories mainly covered age, sex, smoking habits, current medications, body mass index (BMI), CVD, cerebral apoplexy, peripheral arterial disease (PAD), hypertension, and duration of diabetes. Patients’ medical details, including presence of hypertension, hyperlipidemia, and diabetes mellitus, were obtained from patient records. BMI was calculated as weight (kg) divided by the square of height (m). In terms of education level, patients were categorized as unschooled or as having attended secondary school, high school, or university. Diabetes of less than 10 years’ duration was regarded as short-duration diabetes, and 10 years’ duration or more as long-duration diabetes in this study. Additionally, we divided patients into two age groups: an older group (more than 60 years old) and a younger group (60 years old or younger).

### 2.3 Measurement and classification of coronary atherosclerosis

All the included patients underwent a CAC CT scan, which was specified in the informed consent materials. The CAC scan of the heart was rapidly acquired, prospectively electrocardiogram-triggered and without contrast. The CAC score was quantified using the Agatston method, in which the area of calcified atherosclerosis (defined as an area of at least 1 mm^2^ with a CT density >130 Hounsfield units [HU]) is multiplied by a density weighting factor and summed for the entire coronary artery tree using a CT dataset with 2.5 to 3.0 mm slice thickness. According to the recommendations of related guidelines, a CAC score of 0 is considered to represent no identifiable plaque burden; a CAC score of 1–10 to represent a minimal identifiable plaque burden; a CAC score of 11–100 to represent a mild atherosclerotic plaque burden; a CAC score of 101–400 to represent a moderate atherosclerotic plaque burden; and a CAC score of more than 400 to represent an extensive atherosclerotic plaque burden. Although a negative or extremely low calcium score (10 or lower) cannot be taken to fully exclude the presence of coronary atherosclerosis, it is consonant with the absence of a fixed (significant) coronary obstructive lesion (likelihood, 5 to 10% or lower) (35, 36). Since a CAC score of less than 100 indicates a low probability of coronary artery disease and coronary events (35–37), we chose 100 as cut-off point. Thus, all patients were assigned to one of two groups according to CAC score: a normal-to-mild group (0 ≤ score ≤ 100), or a moderate-to-severe group (≥101) (34).

### 2.4 Dental history and oral examination

Two trained examiners performed all oral examinations. All patients were examined with a dental explorer, mouth mirror, and (Williams and WHO) periodontal probe. They all underwent a clinical periodontal test and a panoramic radiograph was taken. The gingival index (GI) was measured according to the Silness and Loe method. Additionally, bleeding on probing (BOP), clinical attachment loss (CAL), calculus index (CI), probing depth (PD), and periodontal index (PI) were measured according to O’Leary’s index around the six Rumford index teeth (38). Periodontitis severity staging (39) was calculated accordingly: for each tooth, the CAL of the most severe site was recorded; a CAL of 1–2 mm (with no tooth loss due to periodontitis) was defined as Stage I, a CAL of 3–4 mm (with no tooth loss due to periodontitis) as Stage II (moderate), a CAL of ≥ 5 mm (with loss of ≤ 4 teeth due to periodontitis) as Stage III, and a CAL of ≥ 5 mm (with loss of ≥ 5 teeth due to periodontitis) as Stage IV (severe). All patients with periodontitis were categorized into three groups (40): a mild periodontitis group (Stage I), a moderate periodontitis group (Stage II), and a severe periodontitis group (Stage III-IV). Tooth loss was assessed via panoramic radiography readings. Extractions of third molars and extractions performed for orthodontic treatment were not considered to represent tooth loss, whereas extractions for the purpose of dental implant treatment were considered to represent tooth loss. All radiography readings and assessments were carried out by one dentist. Tooth loss was categorized as 0 to 9, 10 to 19, or 20 to 31 missing teeth (41).

### 2.5 Sample collection and preparation

Saliva and blood samples were obtained on the same day at the time of the patient’s first visit. After overnight fasting, venous blood samples were collected from all patients. Unstimulated whole buccal saliva was collected from individuals as previously described (42). Briefly, saliva was collected during the course of 3 min after an overnight fast and without prior oral hygiene measures. Saliva samples were immediately frozen at -80°C and stored for less than 3 months before biochemical analysis.

### 2.6 Biochemical measurements

Levels of total cholesterol (TC), low-density lipoprotein cholesterol (LDL-C), high-density lipoprotein cholesterol (HDL-C), triglycerides (TGs), fasting plasma glucose (FPG), glycosylated hemoglobin (HbA1c), and serum creatinine were measured using suitable laboratory techniques. ELISA assay kits supplied by Abcam (Cambridge, MA, USA) were used on saliva samples stored at −80°C to determine interleukin-6 (IL-6), matrix metalloproteinase-9 (MMP-9), and TNF-α levels.

### 2.7 DNA extraction and PCR amplification

Microbial DNA was extracted from saliva samples using the E.Z.N.A.® Soil DNA Kit (Omega Bio-tek, Norcross, GA, U.S.) according to the manufacturer’s protocols. The V1–V9 regions of the bacteria 16S ribosomal RNA gene were amplified by PCR (95°C for 2 min, followed by 27 cycles at 95°C for 30 s, 55°C for 30 s, and 72°C for 60 s and a final extension at 72°C for 5 min) using primers 27F 5′-AGRGTTYGATYMTGGCTCAG-3′ and 1492R 5′-RGYTACCTTGTTACGACTT-3′, where the barcode represents an eight-base sequence unique to each sample. PCR reactions were performed in triplicate using a 20 μL mixture containing 4 μL of 5 × FastPfu Buffer, 2 μL of 2.5 mM dNTPs, 0.8 μL of each primer (5 μM), 0.4 μL of FastPfu Polymerase, and 10 ng of template DNA. Amplicons were extracted from 2% agarose gels and purified using the AxyPrep DNA Gel Extraction Kit (Axygen Biosciences, Union City, CA, U.S.) according to the manufacturer’s instructions.

### 2.8 Library construction and sequencing

SMRTbell libraries were prepared from the amplified DNA by blunt-ligation according to the manufacturer’s instructions (Pacific Biosciences). Purified SMRTbell libraries from the Zymo and HMP mock communities were sequenced on dedicated PacBio Sequel II 8M cells using the Sequencing Kit 2.0 chemistry. Purified SMRTbell libraries from the pooled and barcoded samples were sequenced on a single PacBio Sequel II cell. All amplicon sequencing was performed by Shanghai Biozeron Biotechnology Co. Ltd (Shanghai, China).

### 2.9 PacBio sequencing data analysis

PacBio raw reads were processed using the SMRT Link Analysis software package, version 9.0, to obtain demultiplexed circular consensus sequence (CCS) reads with the following settings: minimum number of passes = 3, minimum predicted accuracy = 0.99. Raw reads were processed through SMRT Portal to filter sequences for length (<800 or >2500 bp) and quality. Sequences were further filtered by removing the barcode, primer sequences, chimeras, and sequences containing 10 consecutive identical bases. OTUs were clustered with 98.65% similarity cut-off using UPARSE (version 7.1; http://drive5.com/uparse/), and chimeric sequences were identified and removed using UCHIME. The phylogenetic affiliation of each 16S rRNA gene sequence was analyzed using RDP Classifier (http://rdp.cme.msu.edu/) against the silva (SSU132)16S rRNA database, with a confidence threshold of 70% (43). A rarefaction analysis based on Mothur v.1.21.1 (44) was conducted to reveal the diversity indices, including the Richness and Shannon diversity indices. A Mann–Whitney U test was performed to assess the statistical significance of differences in diversity indices between samples. Differences were considered significant at p < 0.05. For identification of biomarkers for highly dimensional colonic bacteria, LEfSe (linear discriminant analysis effect size) analysis was conducted (45). The Kruskal–Wallis rank-sum test was performed to examine the changes and dissimilarities among classes, followed by LDA analysis to determine the effect size for each of the distinctively abundant taxa (46).

### 2.10 Statistical analysis

SPSS 21.0 (IBM Corp., Armonk, NY, USA) was used for statistical analysis. Data are expressed in the form mean ± standard deviation (SD) for continuous variables and percentages (%) for categorical variables. Between-group differences were analyzed using a two-tailed Student’s t-test or non-parametric t-test. Categorical variables were compared using the chi-squared test or Fisher’s exact test. The association between the severity of periodontal disease or missing teeth and the presence of moderate-to-severe coronary atherosclerosis was analyzed via **multivariate logistic regression**, adjusted for main effects of all covariates, including age, sex, education level, smoking, LDL, HbA1c, BMI, duration of diabetes, and history of statin treatment. All p-values were two-tailed, and p < 0.05 was considered statistically significant.

## 3 Results

### 3.1 General characteristics

184 patients (92 male and 92 female; mean age, 63 ± 10 years) were allocated to the normal-to-mild group, and 88 (54 male and 34 female; mean age, 65 ± 7 years) to the moderate-to-severe group. Demographical, medical, and biochemical data, including medications, dental habits, and education status, are presented in **Table 1**. Patients in the moderate-to-severe group were older than those in the normal-to-mild group (P <0.001), and male subjects constituted the majority in the moderate-to-severe group (P <0.001). More subjects in the moderate-to-severe group were current smokers (P<0.01). The duration of diabetes was longer in the moderate-to-severe group than in the normal-to-mild group (P<0.001). However, there was no statistically significant difference in HbA1c levels between the two groups. Lower TC and LDL-C levels unexpectedly occurred in the moderate-to-severe group, with the differences compared to the normal-to-mild group being statistically significant (P<0.001). It is noteworthy that the rate of statin use at baseline was also higher in the moderate-to-severe group, which may be the reason for the lower TC and LDL-C levels. Regarding the salivary markers measured, IL-6 and TNF-α levels were significantly higher in the moderate-to-severe group than in the normal-to-mild group, whereas salivary MMP-9 levels did not differ significantly between the groups.

**Table 1:** Characteristics of the subjects, grouped by extent of coronary atherosclerosis.

Characteristics	Normal-to-mild group (n=184)	Moderate-to-severe group (n=88)
Sex		
Female	50%	39%^*^
Male	50%	61%^*^
**Age** (years)	57 ± 11	65 ± 7^*^
**Duration of diabetes** (years)	6.6 ± 6.68	14.2 ± 8.27^*^
Smoking		
Never	67%	38%*
Current smoker	30%	52%*
Quit	3%	10%
**BMI (kg/m^2^)**	23.71	24.27
Education		
Unschooled	17%	13%
Secondary school	53%	55%
High school	18%	19%
University	12%	13%
**Statin use**	15%	37%^*^
Biological data		
HbA1c (%)	9.76 ± 3.12	8.90 ± 3.42
TC (mmol/L)	4.58	3.68^*^
TG (mmol/L)	1.83	1.67
HDL-C (mmol/L)	1.17	1.09
LDL-C (mmol/L)	2.81	2.09^*^
sLDL-C (mmol/L)	430.98	336.25^*^
Salivary IL-6 (pg/ml)	1.74 ± 0.75	3.05 ± 0.49*
Salivary MMP-9 (ng/ml)	437.71 ± 64.78	567.09 ± 31.79
Salivary TNF-α (pg/ml)	11.26 ± 7.01	45.00 ± 13.61*
Frequency of dental visits		
Regular	8.5%	7.2%
Irregular	91.5%	92.8%

Continuous variables are expressed in the form mean ± SD; categorical variables are expressed as percentages.

*p < 0.05 vs. normal-to-mild group.

sLDL-C, small, dense low-density lipoprotein cholesterol.

### 3.2 Dental conditions of patients with T2DM in different coronary atherosclerosis groups

The periodontitis parameters of the subjects are shown in **Table 2**. CAL values were much higher among patients in the moderate-to-severe coronary atherosclerosis group (P=0.05). Additionally, the results revealed that individuals in the moderate-to-severe group had more tooth loss compared to patients in the normal-to-mild group (P=0.016). In analyzing patients’ tooth counts, we observed that more subjects in the normal-to-mild group had more than 25 teeth and 11–24 teeth (P =0.0001, P =0.001, respectively). Severe periodontitis occurred more frequently among patients in the moderate-to-severe coronary atherosclerosis group, and this increase was statistically stronger in male patients (P=0.005). Among female patients, there was an increase in the proportion of moderate periodontitis among patients with moderate-to-severe coronary atherosclerosis, with the difference being statistically significant (P=0.027).

**Table 2:** Periodontal parameters of the subjects.

Oral parameters	Normal-to-mild group (n=184)	Moderate-to-severe group (n=88)	P value
**PI**	1.76 ± 0.24	1.85 ± 0.38	0.168
**PD**	3.02 ± 0.68	3.01 ± 0.65	0.957
**CI**	1.78 ± 0.25	1.82 ± 0.35	0.445
**CAL**	3.51 ± 1.21	3.97 ± 1.06*	0.05
**GI**	2.29 ± 0.34	2.35 ± 0.32	0.404
Number of teeth (n)			
>25	116	33*	0.0001
11-24	61	48*	0.01
<10	7	7	0.248
**Number of teeth lost**	4.99 ± 7.3	8.56 ± 7.54*	0.016
Severity of periodontitis (n)			
Mild	50	15	0.067
Moderate	70	32	0.789
Severe	64	41	0.061
Severity staging of periodontitis for males (n)			
Mild	22	8	0.292
Moderate	33	11	0.054
Severe	38	35*	0.005
Severity of staging periodontitis for females (n)			
Mild	29	6	0.143
Moderate	36	21*	0.027
Severe	26	7	0.368

Continuous variables are expressed in the form mean ± SD; categorical variables are expressed as percentages.

*p < 0.05 vs. normal-to-mild group.

### 3.3 Association of tooth loss and periodontitis parameters with moderate-to-severe diabetic coronary atherosclerosis

The results of univariate logistic regression analyses, with ORs and CIs, are listed in **Table 3** (model 1). CAL (P=0.021) and having 10–19 missing teeth (p=0.01) were found to be highly associated with the presence of moderate-to-severe coronary atherosclerosis. The ORs calculated for CAL and for 10–19 missing teeth were 1.536 (95% CI: 1.066–1.214) and 4.106 (95% CI: 1.404–12.009), respectively. The results of multivariate logistic regression analysis are also presented in **Table 3** (model 2). Adjusting for parameters such as age, sex, education level, smoking, LDL, HbA1c, BMI, duration of diabetes, and history of statin treatment in the multivariate logistic regression, we again observed a correlation with moderate-to-severe coronary atherosclerosis among patients with T2DM for CAL (OR: 1.231; 95% CI: 1.004–1.516, P = 0.047) and for having 10-19 missing teeth (OR: 1.604; 95% CI: 1.393–6.555, P=0.05). Unexpectedly, severity staging of periodontitis was not significantly associated with the presence of moderate-to-severe diabetic coronary atherosclerosis in either the unadjusted or the adjusted model.

**Table 3:** Multivariate logistic regression models of the association of CAL and tooth loss with the presence of moderate or severe coronary atherosclerosis in patients with T2DM.

	Model 1^a^	Model 2^b^
CAL		
OR (95% CI)	1.536 (1.066–1.214)	1.231 (1.004–1.516)
P value	0.021	0.047
Number of teeth lost		
**0–9 missing teeth**		
**10–19 missing teeth**		
OR (95% CI)	4.106 (1.404–12.009)	1.604 (1.393–6.555)
P value	0.01	0.05
20–31 missing teeth		
OR (95% CI)	2.433 (0.626–9.466)	0.875 (0.131–5.834)
P value	0.199	0.89
Severity of periodontitis (n)		
**Mild**		
**Moderate**		
OR (95% CI)	0.362 (0.117–1.122)	1.801 (0.264–12.285)
P value	0.078	0.548
Severe		
OR (95% CI)	0.657 (0.276–1.564)	2.414 (0.674–8.652)
P value	0.342	0.176

^a^unadjusted.

^b^adjusted for age, sex, education level, smoking, LDL, HbA1c, BMI, duration of diabetes, and history of statin treatment.

### 3.4 Association of tooth loss and periodontitis parameters with extent of coronary atherosclerosis in patients with short- vs. long-duration diabetes

When we compared the parameters of periodontitis between patients with different durations of diabetes, we found that CAL values were higher in patients with long-duration diabetes. However, when patients with short-duration diabetes and moderate-to-severe coronary AS were compared to patients with long-duration diabetes and normal-to-mild coronary AS, the inter-group difference in CAL values disappeared (**Table 4**). Additionally, the results revealed that, among patients with short-duration diabetes, individuals in the moderate-to-severe group had more tooth loss compared to those in the normal-to-mild group. However, there was no relationship between tooth loss and extent of coronary AS among patients with long-duration diabetes (**Table 5**).

**Table 4:** Periodontitis parameters and salivary inflammatory markers in patients with short- vs. long-duration diabetes.

	All patients			Sub-groups of specific patients		
	Short-duration diabetes	Long- duration diabetes	P value	Short-duration diabetes with moderate-to-severe AS	Long-duration diabetes with normal-to-mild AS	P value
**PI**	1.71 ± 0.32	1.80 ± 0.32	0.076	1.73 ± 0.40	1.81 ± 0.27	0.453
**PD**	2.91 ± 0.68	3.05 ± 0.66	0.179	2.90 ± 0.91	3.20 ± 0.71	0.194
**CI**	1.71 ± 0.32	1.76 ± 0.36	0.416	1.70 ± 0.31	1.81 ± 0.29	0.305
**CAL**	3.38 ± 1.15	3.87 ± 1.19*	0.011	3.77 ± 0.77	4.00 ± 1.40	0.593
**GI**	2.21 ± 0.38	2.26 ± 0.43	0.398	2.24 ± 0.29	2.35 ± 0.34	0.345
**Number of teeth lost**	4.93 ± 0.721	6.98 ± 0.924	0.083	8.62 ± 2.21	6.57 ± 1.49	0.431
**IL-6 (pg/ml)**	2.49 ± 0.75	2.90 ± 0.46	0.633	2.04 ± 0.74	3.64 ± 5.07	0.368
**MMP-9 (ng/ml)**	513.83 ± 52.35	554.58 ± 32.54	0.509	411.99 ± 82.12	611.91 ± 85.48	0.111
**TNF-α (pg/ml)**	35.50 ± 10.15	43.09 ± 26.29	0.746	23.08 ± 17.94	86.40 ± 77.55	0.474

Continuous variables are expressed in the form mean ± SD.

*p < 0.05 vs. group with short-duration diabetes.

**Table 5:** Periodontitis parameters and salivary inflammatory markers in patients with short- vs. long-duration diabetes and different degrees of coronary atherosclerosis.

	Short duration of diabetes			Long duration of diabetes		
	Normal-to-mild coronary AS	Moderate-to-severe coronary AS	P value	Normal-to-mild coronary AS	Moderate-to-severe coronary AS	P value
**PI**	1.74 ± 0.23	1.73 ± 0.40	0.876	1.81 ± 0.27	1.87 ± 0.40	0.607
**PD**	2.95 ± 0.68	2.90 ± 0.91	0.060	3.20 ± 0.71	3.06 ± 0.69	0.491
**CI**	1.76 ± 0.24	1.70 ± 0.31	0.412	1.81 ± 0.29	1.79 ± 0.44	0.878
**CAL**	3.33 ± 1.11	3.77 ± 0.77	0.172	4.00 ± 1.40	4.04 ± 1.16	0.928
**GI**	2.27 ± 0.33	2.24 ± 0.29	0.800	2.35 ± 0.34	2.36 ± 0.32	0.990
**Number of teeth lost**	4.06 ± 0.86	8.62 ± 2.21*	0.037	6.57 ± 1.49	8.04 ± 1.35	0.472
**IL-6 (pg/ml)**	3.13 ± 0.24	2.04 ± 0.74	0.420	3.64 ± 5.07	5.95 ± 1.49	0.742
**MMP-9 (ng/ml)**	549.66 ± 39.66	411.99 ± 82.12	0.154	611.91 ± 85.48	552.17 ± 95.01	0.656
**TNF-α (pg/ml)**	49.43 ± 16.94	23.08 ± 17.94	0.493	86.40 ± 77.55	41.31 ± 35.26	0.570

Continuous variables are expressed in the form mean ± SD.

*p < 0.05 vs. normal-to-mild coronary AS group.

### 3.5 Association of tooth loss and periodontitis parameters with extent of coronary atherosclerosis in patients of different age groups

The results showed that older patients had higher CAL values than younger patients. However, among younger patients with moderate-to-severe coronary AS, all the parameters of periodontitis and tooth loss were higher than in older patients with normal-to-mild coronary AS, with no statistically significant differences (**Table 6**). Evaluating the results in younger patients with varying degrees of coronary AS, we observed that individuals with moderate-to-severe coronary AS had higher CAL and GI values, as well as more tooth loss, compared to individuals with normal-to-mild coronary AS. However, we did not observe this difference between the normal-to-mild and the moderate-to-severe group among older patients (**Table 7**).

**Table 6:** Periodontitis parameters and salivary inflammatory markers in patients in different age groups.

	All patients			Sub-groups of specific patients		
	Younger group	Older group	P value	Younger group with moderate-to-severe AS	Older group with normal-to-mild AS	P value
**PI**	1.71 ± 0.32	1.80 ± 0.32	0.076	1.94 ± 0.41	1.75 ± 0.24	0.065
**PD**	2.91 ± 0.68	3.05 ± 0.66	0.179	3.32 ± 0.52	3.09 ± 0.69	0.346
**CI**	1.71 ± 0.32	1.76 ± 0.36	0.416	1.94 ± 0.40	1.78 ± 0.26	0.139
**CAL**	3.38 ± 1.15	3.87 ± 1.19*	0.011	4.40 ± 0.81	3.76 ± 0.99	0.077
**GI**	2.21 ± 0.38	2.26 ± 0.43	0.398	2.57 ± 0.37	2.31 ± 0.30**	0.029
**Number of teeth lost**	4.93 ± 7.46	6.98 ± 7.39	0.083	8.80 ± 2.54	6.17 ± 1.23	0.35
**IL-6 (pg/ml)**	4.37 ± 0.55	4.99 ± 1.59	0.764	3.32 ± 0.70	2.93 ± 0.26	0.535
**MMP-9 (ng/ml)**	513.83 ± 52.35	554.58 ± 34.55	0.509	411.60 ± 127.90	569.05 ± 49.46	0.256
**TNF-α (pg/ml)**	35.50 ± 10.15	43.09 ± 26.29	0.746	3.57 ± 2.07	58.51 ± 22.97	0.64

Continuous variables are expressed in the form mean ± SD.

*p < 0.05 vs. younger group.

**p<0.05 vs. younger group with moderate-to-severe AS.

**Table 7:** Periodontitis parameters and salivary inflammatory markers in older and younger patients with different degrees of coronary atherosclerosis.

	Younger patients			Older patients		
	Normal-to-mild coronary AS	Moderate-to-severe coronary AS	P value	Normal-to-mild coronary AS	Moderate-to-severe coronary AS	P value
**PI**	1.76 ± 0.25	1.94 ± 0.41	0.231	1.75 ± 0.24	1.79 ± 0.39	0.654
**PD**	2.94 ± 0.69	3.32 ± 0.52	0.129	3.09 ± 0.69	2.91 ± 0.64	0.276
**CI**	1.77 ± 0.25	1.94 ± 0.40	0.093	1.78 ± 0.26	1.71 ± 0.40	0.351
**CAL**	3.25 ± 1.35	4.40 ± 0.81*	0.017	3.76 ± 0.99	3.82 ± 1.08	0.809
**GI**	2.27 ± 0.37	2.57 ± 0.37*	0.034	2.31 ± 0.30	2.24 ± 0.27	0.348
**Number of teeth lost**	3.28 ± 0.84	8.80 ± 2.54*	0.012	6.17 ± 1.23	8.03 ± 1.29	0.306
**IL-6 (pg/ml)**	3.50 ± 0.47	3.32 ± 0.70	0.855	2.93 ± 0.26	3.02 ± 1.16	0.769
**MMP-9 (ng/ml)**	553.48 ± 52.89	411.60 ± 127.90	0.306	569.05 ± 49.46	518.89 ± 77.47	0.569
**TNF-α (pg/ml)**	54.76 ± 35.75	3.57 ± 2.07	0.548	58.51 ± 22.97	41.62 ± 27.75	0.644

Continuous variables are expressed in the form mean ± SD.

*p < 0.05 vs. normal-to-mild coronary AS group.

### 3.6 CAC scores among T2DM patients with different severity staging degrees of periodontitis

**Figure 1:**
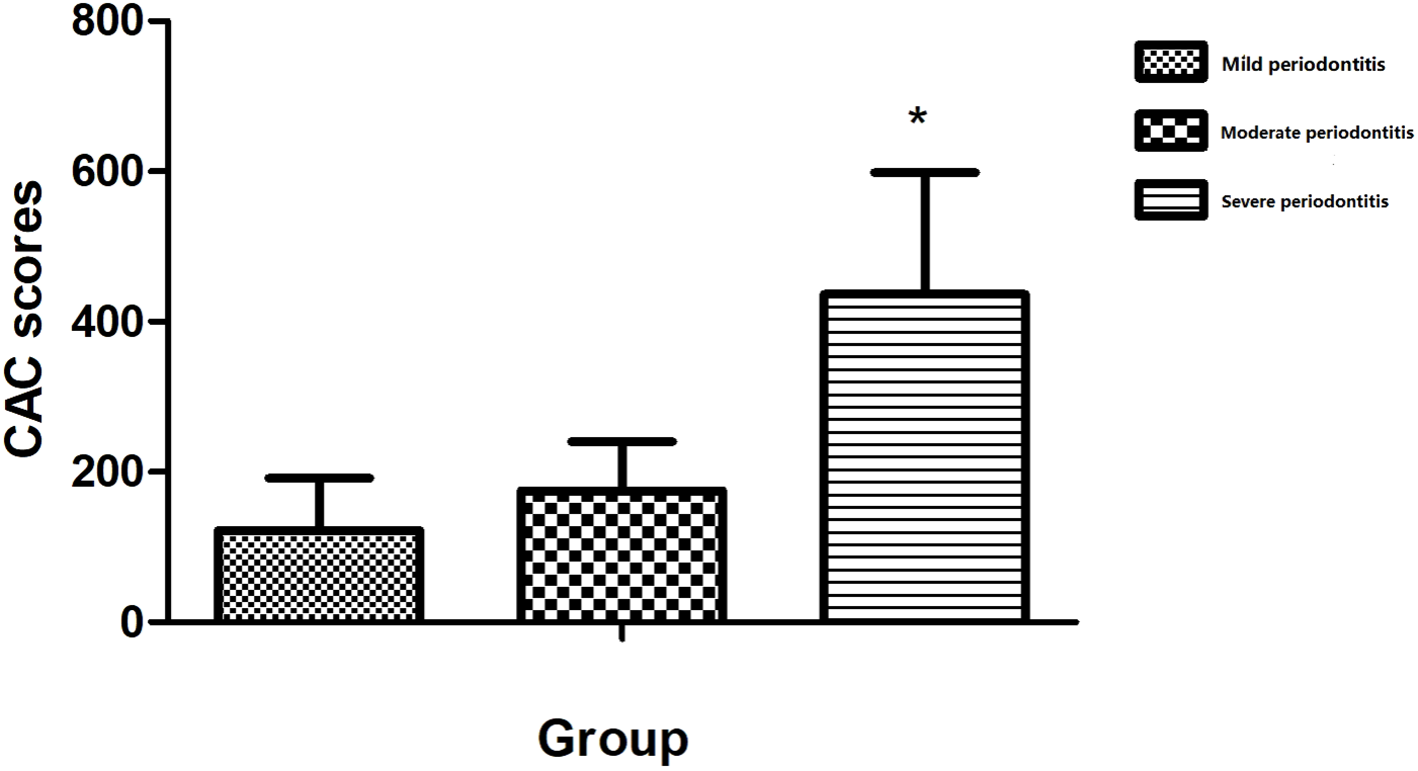
The CAC scores of patients grouped by severity of periodontitis. *p <0.05 vs. the mild group.

The CAC scores of patients in different severity staging groups for periodontitis are presented in **Figure 1**. CAC scores were higher in the severe group (436.17 ± 162.424) than in the mild group (122.3 ± 69.394), with the difference reaching statistical significance (P=0.043).

### 3.7 Difference in distinct microbiota between patients in the normal-to-mild coronary AS group and those in the moderate-to-severe group

**Figure 2:**
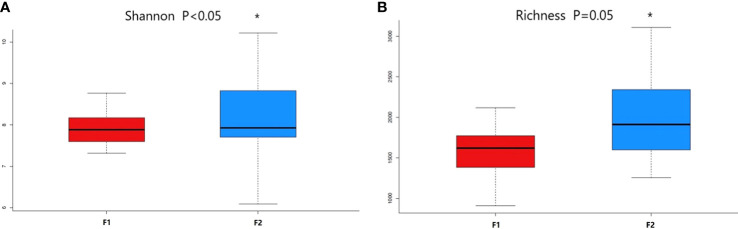
Comparison of alpha diversity metrics of plasma bacterial communities between the normal-to-mild and moderate-to-severe coronary AS groups. **(A)** Boxplots for comparison of species richness between the two study groups (Richness). **(B)** Boxplots for comparison of species diversity (Shannon index). (F1: moderate-to-severe coronary AS group; F2: normal-to-mild coronary AS group). * P≤0.05 vs F1 group.

Alpha diversity indices indicated significant differences in Shannon index and Richness between the two coronary AS groups (p = 0.037 and p = 0.05, respectively), indicating higher abundance and greater diversity in the normal-to-mild group (**Figures 2A, B**).

### 3.8 Distribution of the predominant bacteria at different taxonomic levels

**Figure 3:**
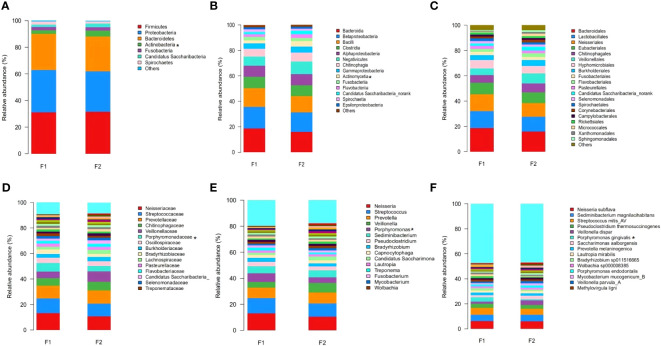
Relative abundance distribution at the level of phylum **(A)**, class **(B)**, order **(C)**, family **(D)**, genus **(E)**, and species **(F)** in the normal-to-mild and moderate-to-severe coronary AS groups. (F1: moderate-to-severe coronary AS group; F2: normal-to-mild coronary AS group.) *p<0.05, *F1* vs. *F2*.

The predominant taxa identified in the dental plaque samples of oral microbiota from the normal-to-mild and moderate-to-severe coronary AS groups at different taxonomic levels are shown in **Figure 3**. At the phylum level, Firmicutes, Proteobacteria, Bacteroidetes, and Actinobacteria accounted for the majority of the total sequences (**Figure 3A**). Levels of Actinobacteria were significantly lower in the moderate-to-severe coronary AS group (p =0.045, Mann–Whitney U test). At the class level, Bacteroidia, Betaproteobacteria, Bacilli, Clostridia, Alphaproteobacteria, Negativicutes, Chitinophagia, Gammaproteobacteria, Actinomycetia, and Fusobacteriia were the ten most common (**Figure 3B**), among which, levels of Actinomycetia were consistently more abundant in the normal-to-mild coronary AS group (p =0.035, Mann–Whitney U test). At the order level, Bacteroidales, Lactobacillales, and Neisseriales were predominant (**Figure 3C**). At the family level, the predominant families were Neisseriaceae, Streptococcaceae, Prevotellaceae, Chitinophagaceae, Veillonellaceae, and Porphyromonadaceae (**Figure 3D**), among which, levels of Porphyromonadaceae were more abundant in the moderate-to-severe coronary AS group (p =0.016, Mann–Whitney U test). At the genus level, Neisseria, Streptococcus, Prevotella, Veillonella, and Porphyromonas were predominant (**Figure 3E**). Porphyromonas levels were significantly higher in the moderate-to-severe coronary AS group (p =0.017, Mann–Whitney U test). Finally, at the species level, the predominant species were Neisseria subflava, Sediminibacterium magnilacihabitans, Streptococcus mitis_AV, Pseudoclostridium thermosuccinogenes, Veillonella dispar, and Porphyromonas gingivalis (**Figure 3F**). Among these, the abundance of Porphyromonas gingivalis differed significantly between the normal-to-mild and the moderate-to-severe coronary AS groups (p =0.042, Mann–Whitney U test). These results indicated distinctive differences in the bacterial communities of the groups with different degrees of coronary AS.

### 3.9 Differentially abundant OTUs in LEfSe analysis

**Figure 4:**
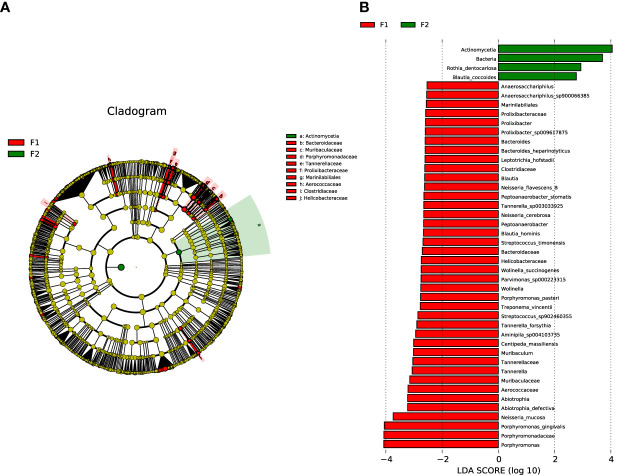
Analysis of species diversity in the normal-to-mild and moderate-to-severe coronary AS groups. **(A)** Cladistic map of microbial evolution. **(B)** Histogram of LDA value distribution. In the evolutionary branching diagram, circles radiating from the inside out indicate taxonomic levels from phylum to genus (or species). Each small circle for a different taxon class represents the taxa of that class, and the diameter of the small circle is proportional to the relative abundance. Species with no significant differences are yellow; red nodes represent the microbiota playing an important role in the moderate-to-severe group; and green nodes represent the microbiota playing an important role in the normal-to-mild group. Bar length in the histogram indicates the size of the impact of different species (LDA score). (F1: moderate-to-severe coronary AS group; F2: normal-to-mild coronary AS group).

To identify the specific bacterial taxa of oral microbiota that were associated with the moderate-to-severe group, we compared the microbial composition of the two groups using the LEfSe method. A cladogram representative of the structure of the oral microbiota and the predominant bacteria is shown in **Figure 4A**, with indications of the largest differences in the taxa represented in the two communities. The levels of members of bacterial taxa belonging to Porphyromonadaceae, Porphyromonas, Porphyromonas gingivalis, and Neisseria_mucosa were increased in the moderate-to-severe coronary AS group, and those of Actinomycetia, Rothia_dentocariosa, and Blautia coccoides were enriched in the normal-to-mild group; thus, these could be used as biomarkers for discrimination between the groups. The LDA bar graph (log 10) for discrimination is presented in **Figure 4B**.

## 4 Discussion

The outcomes of the present study indicated that there exists a significant association of periodontitis and tooth loss with the presence of moderate-to-severe coronary atherosclerosis in patients with T2DM. Notably, we found that the nature of this association varies with sex, age, and duration of diabetes.

A number of other analyses have reported previously on the relationship between periodontitis and CVDs (47, 48). These studies have focused primarily on the risk of myocardial infarction or stroke and the prevalence of coronary heart disease in relation to periodontitis, whereas we have used the extent of atherosclerosis as the primary outcome. To the best of our knowledge, this is also the first study to identify a relationship between periodontitis and the extent of coronary atherosclerosis evaluated by CAC scores in patients with T2DM. Our findings will be beneficial in reducing cardiac deaths in patients with T2DM through the identification of individuals with a higher risk of incident cardiovascular events for early intervention.

Previous reports have similarly supported a relationship between severity staging of periodontitis and cardiovascular disease (13–15, 49) or sub-atherosclerosis (50, 51). In contrast, here we did not observe an association between severity staging of periodontitis and extent of coronary AS. This conflicting result may be due to variation in the criteria used for periodontitis severity staging and cardiovascular disease. Our results also reported sex differences. Our findings suggest that women with moderate periodontitis and men with severe periodontitis should be screened for moderate or severe coronary atherosclerosis in order to prevent cardiovascular events. The sex differences reported here might be attributable to the fact that women may have comparably less severe periodontitis because of their lower smoking rate. Another reason may be that postmenopausal women are prone to coronary AS. A recent publication has reported on sex differences in the relationship of periodontal disease with clinical end-points (52). Therefore, our data support this report with respect to coronary AS in patients with T2DM.

Among all the parameters of periodontitis that we examined in this study, CAL levels merit the most attention. CAL mainly represents the severity of periodontitis, while other parameters (such as PD, PI, and GI, among others) indicate the complexity of management (53). Our results indicated that the absolute value of CAL was more meaningful in indicating the extent of coronary AS in patients with T2DM than other parameters of periodontitis or the severity staging of periodontitis.

Beck et al. emphasized that the cumulative effect of periodontal infection can be evaluated by measuring the number of teeth lost (54). Based on our results, we suggest that tooth loss could be used as a marker of the extent of coronary AS in T2DM by almost any healthcare worker. Our results are consistent with studies showing an association between tooth loss and CVDs (55–58). It was a little unexpected that our study indicated an association between 10–19 missing teeth and moderate-to-severe coronary AS, rather than 20–31 missing teeth. Although this is not currently very clear, it may be attributable to the fact that patients with 20–31 missing teeth may have more confounding factors, such as older age, longer duration of diabetes, or a higher prevalence of smoking or statin use (data not shown), which could affect both periodontitis and CVDs. Our results suggested that particular attention should be paid to CVDs in patients suffering from diabetes with ≥10 missing teeth.

The present study also demonstrated the influence of age and duration of diabetes on the association between periodontitis and coronary AS. Our results indicated that clinicians should pay more attention to CAL levels and missing teeth in younger patients or patients with short duration of diabetes. Age is a risk factor for both periodontitis and coronary AS. When we examined the specific group of younger patients with moderate-to-severe coronary AS, we found periodontal parameters indicating greater severity in comparison to those of older patients with normal and mild coronary AS. Although this difference was not statistically significant, it indicated at least that the younger patients with severe atherosclerosis were not better off in terms of periodontal status than older patients with mild atherosclerosis, despite having the advantage of age. Similar results were observed in patients with short duration of diabetes. Higher levels of CAL or more missing teeth in younger patients or patients with short-duration diabetes may be clues to the presence of moderate or severe coronary AS. We did not observe differences in CAL or number of missing teeth between the groups with different degrees of coronary AS among older patients or patients with long-duration diabetes. This is in accordance with previous reports showing that the association between periodontitis and atherosclerotic changes is stronger in younger people (16, 59, 60) and is sometimes not seen in older subjects (16, 61). One reasonable explanation for this may be the cumulative nature of periodontal disease and tooth loss, such that as subjects age there are fewer teeth available for periodontitis to act on (62).

Recently, there has been growing appreciation that saliva can reflect virtually the entire spectrum of health, from normality to disease states (63). Some saliva markers may be relevant for the prognosis, diagnosis, and management of periodontitis (64) and CVDs (65). The value of salivary biomarkers, such as inflammatory cytokines, ILs and TNFs, enzymes, and growth factors, in the diagnosis and monitoring of gingivitis and periodontitis has been verified (66). Our study also found that salivary IL-6 and TNF-α were higher in patients with T2DM and more severe coronary AS. However, we did not observe a statistically significant difference between groups when we divided patients into groups according to age, duration of diabetes, and extent of coronary AS. The reason for this may be that this study included a small number of participants. The findings of our study also suggested that salivary levels of IL-6 and TNF-α may be biomarkers of moderate-to-severe coronary AS in patients with T2DM. Although these results remain to be validated for clinical application, they suggest that saliva could be used to measure the levels of cardiometabolic risk markers in the near future; they also demonstrate its potential in the identification and management of diseases.

Bacteremia is frequent in periodontitis and provides a pathway for periodontal pathogens to access the arterial wall. Our study indicated that Porphyromonas gingivalis and Neisseria mucosa were increased in patients with diabetes and moderate-to-severe coronary AS. There is extensive evidence that P. gingivalis is particularly important as a potential microbial link between human periodontitis and atherosclerosis, as indicated by the correlation between circulating specific antibodies and risk of stroke (67) and cardiovascular disease (68–70). Our results are in agreement with these reports and demonstrate the important role of P. gingivalis in linking periodontitis and severe coronary atherosclerosis in patients with T2DM. The mechanisms underlying the role of P. gingivalis in connecting the triple axis of periodontitis, CVD, and T2DM are not very clear at present. One possible mechanism can be described as follows. P. gingivalis can increase the accumulation of reactive oxygen species (ROS), leading to oxidative stress (71). Oxidative stress contributes to damage to major cellular components, including DNA, proteins, and lipids. Autoantibodies may cross-react with oxidative epitopes, such as MDA, MAA, and AGE epitopes on sugars, OxLDL, oxPL, and P. gingivalis. These PAMPs and DAMPs may serve as bridges or links between periodontitis and atherosclerosis with T2DM (71, 72). Further research should be conducted to verify this hypothesis. Neisseria mucosa is one of the core bacterial groups in saliva and in supragingival and subgingival plaque (73). However, there has been no report of its role in atherosclerosis. It is not clear whether Neisseria mucosa contributes to the development of AS in patients with diabetes. Further studies should be conducted to determine its role in AS.

This study has several limitations that are inherent to cross-sectional analyses. The relationships reported here should not be interpreted as causal. Our cross-sectional study design lacked information on the time sequence of events and therefore did not permit the identification of causal relationships. Additionally, the sample size of this study was not large enough. In future, cohort studies will be required to determine the correlation between coronary atherosclerosis and periodontal disease based upon well-determined diagnostic criteria and to evaluate the effects of complicating variables for either atherosclerosis or periodontitis. Despite its advantages of being cost-effective and non-contrast, the use of CAC score as the primary outcome measure in this study may impose some limitations. Following important single-center and clinical registry studies, as well as large long-term population-based observational studies, clinical practice guidelines consider CAC scoring to be potentially useful as a way of improving cardiovascular risk assessment in asymptomatic individuals and as a guide for initiation or deferral of preventive therapies (74); however, the National Heart, Lung, and Blood Institute (NHLBI) only endorses its use in the study of subclinical cardiovascular disease. The prevalence of CAC has been found to differ by ethnicity, gender, and age. Taking this into consideration, the use of a CAC score cut-off of ≥75th percentile for age, sex, and race as a validated measure of atherosclerosis may to some extent be more precise than a cut-off value of CAC score ≥ 100 (75).

In conclusion, our data indicate that CAL and number of missing teeth are associated with extent of coronary atherosclerosis, especially in younger patients and patients with short-duration diabetes. Our results also suggest that saliva inflammatory factors may be biomarkers of moderate-to-severe coronary AS in patients with T2DM. Considering that CVDs are the primary cause of mortality and morbidity in patients with T2DM, the association between periodontitis and moderate-to-severe coronary AS may be of major importance and merits assessment as early as possible in dental clinics. Dentists may become more involved in the management of diabetic cardiovascular complications.

## Data availability statement

The datasets presented in this study can be found in online repositories. The names of the repository/repositories and accession number(s) can be found below: https://www.ncbi.nlm.nih.gov/sra/?term=SRP453053. The code can be found here: https://github.com/5798132/xulei.

## Ethics statement

The studies involving humans were approved by Human Research and Ethics Committee of Shanghai East Hospital. The studies were conducted in accordance with the local legislation and institutional requirements. The participants provided their written informed consent to participate in this study.

## Author contributions

BF and LX contributed to the conception and design of the study. HL, XY, and ZL organized the database. MS and ZL performed the oral examinations. ZL performed the ELISA experiments. LX performed the statistical analysis. MS and LX wrote the first draft of the manuscript. All authors contributed to the article and approved the submitted version.
